# Different techniques in transalveolar maxillary sinus elevation: A literature review

**DOI:** 10.34172/japid.2021.004

**Published:** 2021-04-06

**Authors:** Ardeshir Lafzi, Fazele Atarbashi-Moghadam, Reza Amid, Soran Sijanivandi

**Affiliations:** ^1^Department of Periodontics, Dental School, Shahid Beheshti University of Medical Sciences, Tehran, Iran; ^2^Dental Research Center, Research Institute of Dental Sciences, Shahid Beheshti University of Medical Sciences, Tehran, Iran

**Keywords:** Dental implant, Maxillary sinus, Sinus floor augmentation, Transalveolar maxillary sinus elevation

## Abstract

Dental implant treatment in the posterior maxilla encounters bone quality and quantity problems. Sinus elevation is a predictable technique to overcome height deficiency in this area. Transalveolar sinus elevation is a technique that is less invasive and less time-consuming, first introduced for ridges with at least 5 mm of bone height. Many modifications and innovative equipment have been introduced for this technique. This review aimed to explain the modifications of this technique with their indications and benefits.

An exhaustive search in PubMed Central and Scopus electronic databases was performed until December 2020. Articles were selected that introduced new techniques for the transalveolar maxillary sinus approach that had clinical cases with full texts available in the English language. Finally, twenty-six articles were included. The data were categorized and discussed in five groups, including expansion-based techniques, drill-based techniques, hydraulic pressure techniques, piezoelectric surgery, and balloon techniques.

The operator’s choice for transalveolar approach techniques for sinus floor elevation can be based on the clinician’s skill, bone volume, and access to equipment. If possible, a technique with simultaneous implant placement should be preferred.

## Introduction

The dental implant is a successful treatment modality used worldwide for functional and esthetic oral rehabilitation.^1^ The posterior maxilla has a unique condition in terms of bone quantity and quality. After tooth extraction, alveolar ridge resorption on the one hand and the pneumatization of the maxillary sinus, on the other hand, cause a deficiency in alveolar bone height, which is a problem for implant placement.^2^ Long-standing edentulism, trauma, developmental disorders, and periodontal diseases are other causes of bone loss in this segment.^3^ Therefore, clinicians might choose to use short dental implants, guided bone regeneration, bone blocks, or sinus floor elevation.^4,5^ Residual volume of the bone, crest morphology, and available space for the prosthesis affect the treatment plan.^3^ There are two major approaches for the elevation of the sinus floor: lateral and transalveolar approaches.^6^ Although the lateral approach has been widely used and proved to be a highly predictable technique,^6,7^ it causes many postoperative complications and patient morbidity.^8^ The main complications with the lateral window approach include Schneiderian membrane perforation, bleeding, implant displacement into the sinus, sinusitis, damage to the adjacent teeth, and ostium obstruction.^8,9^ The implants could be placed right after the sinus floor elevation (simultaneous) or after the initial healing of the prepared site (staged).^7^

The crestal approach was first suggested by Tatum, who used a “socket former” to create a “greenstick fracture” in the sinus floor.^10^ After that, Summers introduced osteotome sinus floor elevation when the residual bone was of poor quality with a height >5 mm.^11^ Then, he added a bone graft to the osteotomy site.^12^ In the less invasive and less time-consuming crestal approach, simultaneous implant insertion has been recommended;^11,13^ however, many complications like postoperative headaches, vertigo, and inner ear injuries still exist.^14^ Many modifications have been introduced for this technique over the years. This study aimed to assess other modifications of closed sinus augmentation besides Summer’s technique.

## Methods

An exhaustive search in PubMed Central and Scopus electronic databases was performed until December 2020 using the following query: (crestal OR closed OR indirect OR transalveolar) AND (maxillary sinus) AND (elevation OR lift OR augmentation). A manual search was also carried out in the bibliography of the selected articles.

The focus question of the study was “what transalveolar maxillary sinus elevation approaches have been introduced for patients in need of dental implant in the posterior maxilla with bone height deficiency” and “what are these methods’ efficacy.”

Eligibility criteria included publications introducing new techniques for crestal maxillary sinus approach that had clinical cases with full text available in the English language. Publications reporting technical keynotes or animal and cadaver studies were excluded. Other innovations, such as controlling techniques with an endoscope, computer-guided methods, or sinus elevation in a fresh socket, were excluded.

The titles and abstracts were read. The full texts of the articles that appeared to meet the inclusion criteria or had insufficient data in the title and abstract were also assessed. The full texts of all the selected articles were reviewed by two authors. The year of publication, the new technique details, instruments, number of patients, patient’s sex and age, average bone height before the procedure, stages of the procedure, use of bone grafts, grafting materials, follow up times, advantages, and disadvantages were extracted from articles and categorized in the tables.

## Results

Totally, 1327 articles from the PubMed and Scopus searches were assessed. Finally, 26 articles were included in the review. In this systematic review, transalveolar sinus elevation techniques were categorized and discussed in the following groups: expansion-based techniques, drill-based techniques, hydraulic pressure techniques, piezoelectric surgery, balloon techniques.

### Expansion-based techniques (Osteotome /Expander)

A summary of studies on expansion-based techniques is presented in Table 1. Davarpanah et al^3^ suggested standard drilling up to 1 mm from the sinus floor, followed by using a resorbable graft material in the site before osteotome placement as a shock absorber. This technique is suitable in areas with dense bone. Drew et al^15^ recommended using a surgical guide and countersink/pilot drills between osteotomes to prevent deviation from the desired path.

**Table 1:** Expansion-based techniques (osteotome/expander) in transalveolar maxillary sinus elevation

**No**	**Reference**	**Stages**	**Patients** **N (M/F)**	**Mean age** **(years)**	**Initial bone** **height (mm)**	**Follow-up** **time (months)**	**Outcome**
1	Davarpanah et al^3^ (2001)	1	NM	NM	NM	NM	• Reduction in operative time • Postoperative comfort
2	Fugazzotto^16^ (2001)	2	61 (26/35)	46-79	NM	36	• 3.92% of sites needed repeated augmentation • Used in atrophic bone (4-5mm height) • Less traumatic than malleting • Saving bone
3	Toffler^17^ (2002)	2	43 (23/20)	56	<6 (Mean: 3.2)	3-35 (mean 15.5)	• Implants with10-13mm length were inserted • Healing time of 5-7 months • 5.47% membrane tear • Nose bleeding • Membrane exposure • Used in atrophic bone (<6mm height)
4	Winter et al ^22^ (2003)	1	20 implant	NM	Mean: 2 mm	12	• Implant with 10-13mm length were inserted • 90% success rate
5	Drew et al^15^ (2007)	1	2 (1/1)	56.5	NM	NM	• In sites with a significant vertical depth
6	Pontez et al^20^ (2010)	1	1M	37	7	24	• Implant with 15 mm height inserted • Weaken the impact of sinus cortical fracture
7	Soltan et al^18^ (2012)	2	1M	75	2	NM	• Implant with 11 mm height inserted • Not recommended if primary • Closure was not achieved • Cost effective • Used in severe atrophic ridge • Less postoperative morbidity because of smaller flap design and minimal osteotomy
8	Isidro et al^19^(2015)	2	33	55.5	Mean: 4.05 ±2.28	72	• No postoperative complication • One graft failure before implant placement • One implant loss • 10 years was 97% • Additional Summers technique may be needed at implant placement
9	Trombelli et al^21^ (2015)	2	3 (2/1)	54.33	Range:2-3	36	• 8 mm implant placed • Histologic confirmation • Used in severe atrophic ridge (2-3 mm)
10	Wang^23^ (2016)	2	1	52	Range:1-2	12	• 10 mm implant placed • Healing 9 months • Used in in severe atrophic ridge (1-2 mm)
11	Kadkhodazdeh et al ^24^ (2020)	1	44 (18/26)	NM	Mean: 8.28 ± 1.58 (premolar site) 7.32 ± 1.43 (molar site)	24 - 60	• 8-12 mm implant placed • Minimal invasion • Time-consuming

NM=not mentioned

Fugazzotto^16^ used a trephine to remove and conserve bone up to the sinus floor and push the bone with an osteotome into the sinus, 1 mm less than the trephine cut. The site was then filled with bone graft material and covered with a membrane. The implant was inserted after the healing time had passed.^16^ The largest trephine without compressing buccal and palatal bone walls was selected. The disadvantage of this two-stage technique was that the height of the augmented bone was less than twice the height of primary residual bone, and if a longer implant was desired, the osteotome and trephine technique had to be repeated.^16^ Toffler^17^ in 2002 named this technique the crestal core elevation. He used hollow core osteotomes and core elevators for elevation after preparing the site with a trephine. In this technique, the attachment of the core with the membrane was preserved. He claimed that this technique reduced membrane perforations.^17^

Soltan et al^18^ suggested using a resorbable StemVie post through a crestal ridge in severe atrophic ridges with difficult access for lateral approach. This technique had two stages, but an implant could be placed simultaneously when sufficient bone was present for stabilization.^18^ Isidro et al^19^ harvested autogenous bone using a trephine bur or cylindrical allograft with 2 mm more diameter and length than the planned implant. The conical-shaped bone graft was then placed at the top of the crest. No micro-screws were used to attach the bone grafts, no particulate bone was packed, and the surgical site was not covered by any sheets. Due to the large size of the graft in this technique, the recommended graft material was allograft. Ramus and the symphysis were used to harvest small blocks of autogenous graft material. The implant was placed after the healing period had passed in the second stage.^19^

Pontes et al^20^ used a connective tissue graft between the sinus floor and osteotome to weaken the impact of sinus cortical fracture and prevent membrane perforation. Trombelli et al^21^ interposed a 3D collagen matrix or graft material between the sinus floor and the osteotome before fracturing the sinus floor. Then, gradual increments of graft material were pushed in using a calibrated osteotome Smart Lift elevator.^21^

Winter et al^22^ described the crestal approach with simultaneous implant placement in severely atrophic ridges. They outlined the rectangular window and then raised the sinus membrane using osteotomes. After sinus elevation, they placed an implant wider than the osteotomy site to gain primary stability. If the primary stability was not achieved, the implant was removed, and a bone graft was added to the site for delayed implant placement. They called this technique sinus/alveolar crest tenting.^22^ Wang et al^23^ described a transcrestal window for 1-2-mm residual alveolar ridges. The window on the crestal bone was prepared by piezoelectric surgery. Then the island bone was penetrated using an osteotome, and a sinus elevator was used to detach and elevate the membrane.^23^

Recently, Kadkhodazadeh et al^24^ introduced the “vertically expander screw” (VES) technique using a threaded expander. In this approach, the initial drilling was performed up to 1 mm from the sinus floor. Then, a threaded expander was used to widen the hole and push the sinus floor up in the vertical direction. Finally, the intended height and width of the prepared site were achieved by a gradual increase in the expander screw’s size.

### Drill-based techniques

A summary of studies on drill-based techniques is given in Table 2. Cosci and Luccioli^25^ introduced a new technique in 2000 using special lifting drills (Fresissima-Torino, Italy) for grinding the sinus floor and membrane elevation. These sequential drills had a small cutting angle of 30º with a built-in water flow system. They claimed that this technique was safe because the sinus floor was perforated, not fractured.^25^ Lozada et al,^26^ in a case report, presented a dome-shaped Dask drill (3.3 mm, Dentium, Korea) for removing the sinus floor bone after standard drilling up to 1 mm from the sinus floor. Then they used a crestal sinus curette (Dentium, Korea) for the complete displacement of the Schneiderian membrane. S-reamer (SCA kit, Neobiotech) was an S-shaped blade-like drill, claimed not to perforate the sinus membrane even after touching it because bone chips in the tip of the drill would prevent membrane perforation.^14^ Jang et al^27^ suggested a rotary-grind bur (RGB) (including reamer or sinus bur) in residual bone heights <4 mm. This system had a stopper with 1-mm increments, which was particularly useful when bone height was insufficient. For cases in which decortication of the sinus floor could not be achieved, an additional DASK drill was used.^27^

**Table 2:** Transalveolar maxillary sinus elevation using drill-based techniques

**No**	**Reference**	**stages**	**Patients** **N (M/F)**	**Mean age** ** (years)**	**Initial bone height (mm)**	**Follow-up time (months)**	**Outcome**
1	Cosci & Luccioli^25^ (2000)	1	237	NM	Range: 4-10	72	• Implant with 13 or 15 mm height inserted • 97% success rate • Omit the malleting • ↓ Risk accidental membrane laceration
2	Lozada et al^26^ (2011)	1	NM	NM	NM	6	• Successful implant placement without postoperative complications • 2 small membrane perforation Facilitates and simplifies the detachment of the membrane
3	Kim et al^14^ (2017)	1	19 (10/9)	49.5	Range: 4-7.8 (mean 6.2)	45.4	• Bone height 8-16.2 mm (mean 12mm) after surgery • 93.5%. success rate • 94.7% success in 5 mm or greater initial bone • 73.3% success rate in less than 4mm initial bone • Noticeable reduction in perforation risk • Rapid surgical performance
4	Jang et al^27^ (2018)	1	10 (3/7)	54.2	Range:2.37–3.82 (mean: 3.41 ± 0.53)	12.0 ± 9.4	• Implant placement on a residual bone height of <4 mm via the crestal approach • No perforation

NM=not mentioned

### Hydraulic pressure techniques

The studies on hydraulic pressure techniques are summarized in Table 3. Chen and Cha^28^ described a method using a 2-mm round bur for tapping the sinus floor and then inflating the Schneiderian membrane by a consistent hydraulic pressure, which was delivered via the pinhole provided by the handpiece. The same pinhole was then used to deliver the bone graft mixture using a 3-mm sinus condenser. The surgeon then used a regular 3-mm drill to create a 2-mm conical shape.^28^

**Table 3:** Transalveolar maxillary sinus elevation using hydraulic pressure techniques

**No**	**Reference**	**Stages**	**Patients** **N (M/F)**	**Mean age (years)**	**Initial** **bone height**	**Follow-up** ** time (months)**	**Outcome**
1	Sotirakis & Gonshor^29^ (2005)	1	11 (5/6)	50	Mean; 4	2-30	• Simple and fast • Minimal postoperative • symptoms
2	Bensaha^30^ (2011)	1	25 (13/12)	40.2 ± 6.5	Mean: 3.9 ± 1.2	2	• 12–15 mm lift was in patients with less than 2 mm of cortical bone available • Predictable procedure with a low complication rate, compared with the lateral approach with piezoelectric surgery.
3	Kao & DeHaven^31^ (2011)	1	1M	65	6.5	3	• 11 mm implant was inserted • The hydrostatic pressure is under careful control of the surgeon and constantly, monitored by pressure meter to avoid the excess pressure
4	Jesh et al^32^ (2013)	1	20 (7/11)	51 ±16	Mean: 4.6 ± 1.4	18	• 95% success rate • 5% minor perforation rate (without complication for implant placement) • A height gain of 9.2-1.7 mm • High patient satisfaction
5	Better et al^34^ (2014)	1	18 (8/10)	52	Mean: 5.5	6-9	• The mean bone height gain was 11.2 mm • Safe and effective
6	Lopez et al^33^ (2014)		40 (14/26)	49.47	Mean: 6-9	12	• Mean membrane elevation was 5.5 mm • Low trauma • High accuracy • Reduced handling of biomaterial, which reduces the risk for contamination

NM=not mentioned

In a hydraulic pressure method used by Sotirakis and Gonshor,^29^ sinus floor fracturing and site preparation were carried out using Summer’s technique. Simultaneous detachment and elevation of the Schneiderian membrane were then achieved by saline solution injection using a modified syringe.^29^

Later, Bensaha^30^ used a water lift system consisting of two different components, an intraosseous small titanium screw used for bone anchorage and a hermetic connector, which injected the liquid through the intraosseous element. He also presented the use of this device with the flapless technique. Kao and DeHaven^31^ also introduced a new device (the Luer-Loc cannula with tapered plug-in end) to create hydraulic pressure. Jesch^32^ introduced the Jeder system consisting of a Jeder drill, a Jeder pump, and a connecting tube set. In this system, the remaining bone was slightly perforated, and then the Jeder pump was used to push the sinus membrane by generating hydraulic pressure and vibrations (1.5 bar). The pressure and volume of the liquid were constantly monitored. The device was controlled with a foot pedal. All the procedures were carried out by a handpiece.^32^

In the technique described by Lopez,^33^ perforation of the sinus floor with any methods was possible (with the clinician’s preference). Then the Hydro-mab kit (HYD-01, FMD, Rome, Italy) was used for membrane elevation. First, a cylindrical ML Dispenser was preloaded with the graft material and screwed into the prepared implant site. Then it was connected to the Hydro-mab, and the graft material (0.5 to 1 mL) was gradually injected in 3-5 minutes. After the removal of the ML Dispenser, the implant tunnel was enlarged if necessary, and the implant fixture was inserted.^33^

Better et al^34^ recommended a kind of implant with an internal channel system (iRaise). An internal channel was present in this self-tapping endosseous implant that allowed gentle injection of 0.9% sterile saline solution (2-3 mL based on the required elevation) into the sinus via the provided tubing port. The saline solution was then retracted using the syringe. Flowable bone graft material was then injected through the same channel. Implant insertion was achieved by osteotomy into the bone graft.^34^

### Piezoelectric surgery

The articles on piezoelectric surgery technique are summarized in Table 4. Fu^35^ in 2010 described the use of piezoelectric surgery for crestal sinus floor elevation. Although they still used an osteotome for fracturing the sinus floor, it was performed with the lightest possible force and minimal malleting. Marchetti et al^36^ used the conventional drill for implant site preparation, and then hard tissue-like bone was cut and abraded by ultrasonic tips vibrating at 24,000 to 29,000 Hz. Ultrasonic tips were used to avoid any damage to the soft tissue like the Schneiderian membrane. The lift of the sinus membrane was performed using a round-headed instrument, which helped prevent lacerations. Before placing the graft mixture in the socket, an equine collagen sponge was placed in the socket to provide additional protection (Antema, Molteni Dental, Milan, Italy).^36^ Baldi^37^ used piezoelectric surgery to remove the sinus floor, and the device had contact with the membrane. The membrane was elevated with a grafting procedure.

**Table 4:** Transalveolar maxillary sinus elevation using piezoelectric surgery

**No**	**Reference**	**stages**	**Patients** **N (M/F)**	**Mean age (years)**	**Initial bone height**	**Follow up time**	**outcome**
1	Fu^35^ (2010)	1	NM	NM	>5mm	NM	• Average bone height increase of 3.5 mm • Least chance of perforation • Patient is more comfortable
2	Marchetti et al^36^ (2010)	1	10(4/6)	43 ± 8.99	Range: 5-6	12	• Average bone augmentation of 4.2 ± 0.98 • Reduce the incidence of perforations of the sinus membrane and in particular to avoid trauma from osteotome malleting
3	Baldi^37^ (2011)	1	16	NM	Mean: 5.48	At least 12	• The mean elevation of the sinus membrane was 6.989 mm, 1 implant failure • Work directly in contact with the sinus membrane, gentle sectioning of bone without damage • More comfortable implant site preparation (Anecdotal reports)

NM=not mentioned

### Membrane balloon elevation technique

The articles on membrane balloon elevation technique are summarized in Table 5. Kfir et al^38^ introduced a device for the ballooning technique via the crestal approach. In this technique, site osteotomy was undertaken following Summer’s technique. After performing the Valsalva maneuver, a gel was injected for lubrication. A metal sleeve (2.6 mm in internal diameter) was screwed in up to 0.5 mm superior to the sinus floor, and an inflatable balloon was advanced 1‒2 mm beyond the tip of the metal sleeve. There was a locking mechanism at the proximal part of the sleeve for anchoring the balloon. The inflator syringe passed a diluted contrast medium (50% Ultravist 370, diluted with normal saline solution) which slowly inflated the balloon. The inflating pressure had to remain <2 atmospheric pressure. After the elevation of the sinus membrane, the balloon was deflated and removed with the sleeve.^38^ The dental implant was inserted simultaneously. Mazor et al used this technique with a flapless approach.^39^

**Table 5:** Transalveolar maxillary sinus elevation using balloon techniques

**No**	**Reference**	**stages**	**Patients** **N (M/F)**	**Mean age** ** (years)**	**Initial bone height**	**Follow-up time**	**Outcome** **(success rate)**
1	Kfir et al^38^(2006)	1	24 (12 /12)	42 ± 9	Mean: 3.7 ± 1.4 Mean: 3.5 ± 1.3	At least 6	• 95.83% success rate • Short learning curve • Not time consuming
2	Mazor et al^39^ (2011)	1	20	49	Range: 2-6	18	• 100% success rate • Minimal postoperative symptoms, reduced patient trauma, improved patient comfort and recuperation, decreased surgical time, faster soft tissue healing, normal oral hygiene procedures immediately post-surgery.

## Discussion

Transalveolar maxillary sinus elevation methods are preferred to the lateral window technique because of less flap reflection and fewer complications such as membrane perforation and bleeding, but case selection remains a crucial point.^8,9^ After the standard Summer’s technique for transalveolar approach,^11^ several modifications have been described to improve the shortcomings of this technique, which are described in detail in this review. One aim of all these techniques is to reduce or eliminate hammering and consequently decrease the patients’ discomfort. Osteotome-based techniques, which still use osteotomes for sinus floor fracture, mitigate this problem,^3,16,17,20,21^ but other techniques eliminate this step.^14,24,25,26,29,37,38^ Randomized clinical trials that compared the Summer’s with Cosci technique showed the success of both methods, but the Cosci technique, which uses a drill for sinus floor perforation, is less time-consuming, decreases intra- and postoperative morbidity, and is associated with more patient satisfaction and preference.^6,40^ Esposito et al,^41^ in a systematic review, concluded that trials comparing different sinus elevation techniques could not suggest one ideal procedure that decreases prosthetic or implant failures. However, patients prefer rotary instruments for crestal sinus lifts over hand malleting. Another problem with an osteotome is poor operative control of greenstick fracture; the techniques that replaced the osteotome step with other devices can overcome this problem.^25^ Standard Summer’s technique needs >5 mm residual bone height. However, the success of Cosci’s technique is confirmed in bones with a height of <5 mm.^42,43^

The presence of septa is another problem when using osteotomes, claimed to be eliminated in Cosci’s and Ballon techniques.^25,44^ In the presence of highly atrophic ridge, lateral window approach or zygomatic implant is the primary choice for dental rehabilitation.^6^ However, some authors still prefer the crestal approach^18,19,23^ because of its less invasiveness and better access.

Piezoelectric-mediated sinus elevation earned the benefits of the piezosurgery device, which only cuts the mineralized structures without damaging the adjacent soft tissues.^37^ Using this property, it has been shown that removing the sinus floor with this device is safe even when in contact with the sinus membrane.^37^ It has been demonstrated that piezoelectric-mediated sinus elevation reduces the membrane perforation rate.^45^ Other benefits of the piezosurgery device include precise cutting, clean and clear surgical site, and more intraoperative control.^37^

Sinus membrane elevation with hydraulic pressure helps the clinician avoid large flap retractions for the lateral approach.^29^ With improvements in equipment for this technique, the hydraulic pressure is distributed to the membrane evenly; therefore, this technique is safe, and the amount of sinus lifting is more than the conventional method.^46^ Balloon elevation technique is mainly used for single-tooth replacements and can be used in bone heights <4 mm and enables a predictable membrane elevation for placing implants measuring 13 mm in length.^39^ Asmael,^47^ in a systematic review of balloon elevation via crestal approach, reported a success rate range of 71.4‒100% (mean 91.6%) and membrane perforation rate of 6.76%. A bone gain of >10 mm was reported. They concluded that this approach has the benefits of the lateral window with minimal invasion.^47^ This technique was also successful in sinuses with septa.^44^ This technique requires considerable skills and equipment and might result in membrane tear.^39^ Mazor et al^39^ concluded that if the initial height is <4 mm, this method is inferior to the lateral window approach.

## Conclusion

In conclusion, many techniques are available for the crestal approach of sinus floor elevation. The clinician can choose one of these techniques based on his/her skill, bone volume, and access to equipment. If possible, a technique with simultaneous implant placement is preferred.

## Authors’ contributions

Concept was explained by AL. The review design and RA & FAM. The manuscript was drafted by FAM & SS and critically revised by AL & RA. All authors have read and approved the final manuscript.

## Availability of data

The data from the reported study are available upon request from the corresponding author.

## Ethics approval

Not applicable.

## Competing interests

The authors declare that they have no competing interests related to authorship and/or publication of this work.
